# Optimizing Tedizolid Dosing in Cerebral Nocardiosis: Clinical Impact of Direct Unbound Concentration Measurement and Population PK Modelling in Two Cases

**DOI:** 10.1093/jacamr/dlag004

**Published:** 2026-01-29

**Authors:** Marc-Olivier Vareil, Margaux Bouet, David Leyssene, Anne-Christine Jaouen, Heidi Wille, Christophe Adier, Laure Alleman, Alexia Chauzy

**Affiliations:** Infectious Diseases Department, Centre Hospitalier de la Côte Basque, Bayonne, France; Infectious Diseases Department, Centre Hospitalier de la Côte Basque, Bayonne, France; Service de médecine interne, Centre Hospitalier d’Arcachon, Arcachon, France; Microbiology Laboratory, Centre Hospitalier de la Côte Basque, Bayonne, France; Microbiology Laboratory, Centre Hospitalier de la Côte Basque, Bayonne, France; Infectious Diseases Department, Centre Hospitalier de la Côte Basque, Bayonne, France; INSERM U1070, Pharmacologie des Anti-infectieux et Antibiorésistance, Poitiers, France; Université de Poitiers, UFR de Médecine- Pharmacie, Poitiers, France; Infectious Diseases Department, Centre Hospitalier de la Côte Basque, Bayonne, France; INSERM U1070, Pharmacologie des Anti-infectieux et Antibiorésistance, Poitiers, France; Université de Poitiers, UFR de Médecine- Pharmacie, Poitiers, France

## Abstract

**Introduction:**

Tedizolid, a second-generation oxazolidinone, exhibits potent *in vitro* activity against Gram-positive bacteria, including Nocardia species, and has a more favourable safety profile than linezolid during prolonged use. However, data on itsCSF penetration and efficacy remain scarce. We describe two cases of *Nocardia farcinica* brain abscess treated with tedizolid and report measured serum and cerebrospinal fluid (CSF) exposures.

**Case reports:**

Two patients with *N. farcinica* brain abscesses (MIC for tedizolid 0.75 mg/L) treated with tedizolid as part of combination therapy. Total and unbound concentrations in serum and CSF were quantified using LC-MS/MS, and PK/PD modelling was performed. In case 1, a 60-year-old man with idiopathic CD4 lymphocytopenia initially improved but relapsed while receiving tedizolid 200 mg once daily. The unbound plasma fraction was 15.7%, and CSF exposure remained low, with a predicted fAUC0–24/MIC <3: below the PK/PD threshold used for staphylococcal skin infections. Tedizolid was discontinued, and the patient subsequently died. In case 2, a 72-year-old diabetic patient received 200 mg twice daily. The unbound plasma fraction was higher (30.1%). PK/PD modelling predicted a CSF fAUC0–24/MIC of 7.5, exceeding the proposed efficacy threshold. The patient completed therapy successfully and remained relapse-free after 2 years.

**Discussion:**

These cases highlight moderate CSF penetration of tedizolid and substantial interpatient variability in protein binding. Direct measurement of unbound concentrations was critical for accurate PK/PD assessment. Although higher dosing may improve central nervous system (CNS) exposure and outcomes, tedizolid should not be considered interchangeable with linezolid for CNS nocardiosis. Individualized monitoring of free plasma levels may help optimize dosing strategies.

## Introduction

Tedizolid, a second-generation oxazolidinone, approved for the treatment of skin and soft tissue infections,^1,2^ exhibits potent activity against Gram-positive pathogens, including methicillin-resistant *Staphylococcus aureus*, *Enterococcus faecalis* and other organisms such as mycobacteria and Nocardia species.^3^

Compared with linezolid, tedizolid display a lower minimum inhibitory concentration (MIC), particularly for *S. aureus* (0.5 mg/L versus 4 mg/L).^4^ Tedizolid also offers a more favourable safety and drug interaction profile, especially regarding the risk of thrombocytopenia during prolonged use.^5–8^

While linezolid is known to achieve high cerebrospinal fluid (CSF) concentrations and is an established option for certain central nervous system (CNS) infections, including cerebral nocardiosis,^9^ data regarding tedizolid’s CSF penetration and clinical efficacy in CNS nocardiosis infections remain scarce.^10,11^

Pharmacologically, tedizolid differs from linezolid by its higher protein binding (70%–90% versus 30%), longer half-life (11 versus 5 hours) and predominant faecal excretion.^12^ Both drugs have high oral bioavailability. The optimal pharmacokinetic/pharmacodynamic (PK/PD) index for oxazolidinone efficacy—and therefore tedizolid—is defined as the ratio of the free 24-hour area under the concentration–time curve (fAUC0–24/MIC) to the minimum inhibitory concentration.^1,13^

Here, we report two cases of brain infections due to *Nocardia farcinica* in which tedizolid was part of the treatment regimen.

## Case reports

### Case 1

A 60-year-old man with idiopathic CD4T-lymphocytopenia and no other underlying conditions presented with disseminated nocardiosis affecting the skin, kidneys, lungs and brain. *N. farcinica* was isolated from blood and brain biopsy, with susceptibility to imipenem, linezolid and tedizolid (MIC for tedizolid: 0.75 mg/L).

Initial therapy included imipenem, linezolid and amikacin for 2 weeks, then imipenem/linezolid. Owing to thrombocytopenia and clinical improvement, the regimen was switched to oral tedizolid (200 mg/day) and cotrimoxazole (800 mg three times daily) on discharge. Considering the patient’s clinical deterioration, an Ommaya reservoir was implanted both to facilitate CSF removal due to intracranial hypertension identified on imaging (10 to 15 mL daily) and to enable intrathecal administration of amikacin (30 mg). The rest of the antibiotic regimen included intravenous imipenem (2 g every 8 hours), cotrimoxazole (1200/480 mg every 6 hours) and intravenous amikacin 850 mg/day, plus oral tedizolid (200 mg). The Ommaya reservoir also allowed for sequential pharmacological sampling of the CSF.

At steady state, plasma (through venous puncture) and CSF samples (via the Ommaya reservoir) were collected immediately before then at 3, 5, 12 and 24 hours after dosing. Ultrafiltrates were prepared after plasma centrifugation (Centrifree^®^; Millipore Merck, Guyancourt, France). Tedizolid concentrations in plasma, ultrafiltrates and CSF were measured using a validated LC-MS/MS assay, slightly adapted from two previously published methods.^14,15^ The standard curve ranged from 5 to 5000 ng/mL, and no samples were below the lower limit of quantification (5 ng/mL). Intra-day and inter-day variability were assessed at three concentration levels (15, 500 and 3750 ng/mL) with precisions <14% and 15%, and bias <14% and 13%, respectively.

A compartmental PK analysis was performed using NONMEM software version 7.4 (ICON, Gaithersburg, MD, USA). A previously published population PK model for tedizolid was adapted to describe plasma and CSF concentrations.^1^ A two-compartment model with sigmoidal absorption and linear elimination described plasma PK; absorption parameters were fixed due to lack of early data, and bioavailability was assumed to be 100%.^16^ Plasma PK parameters were similar to published data, except for a shorter half-life (6.3 h versus 11.2 h in healthy volunteers). CSF data were best described by a one-compartment model with a fixed CSF volume (Figure S1, available as Supplementary data at *JAC-AMR* Online).^17^ Residual variability was modelled as proportional error for all matrices. All final model parameters were estimated with good precision (Table S1).

**Figure 1.**
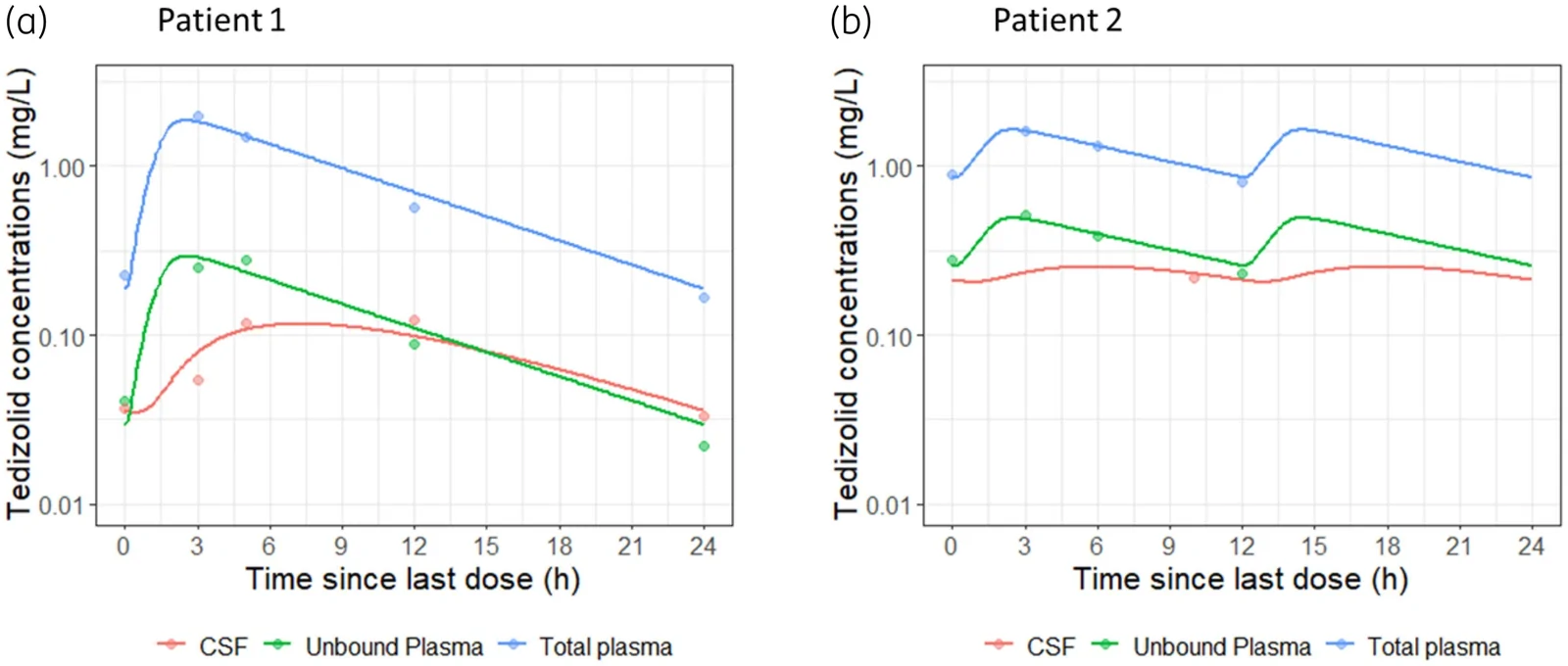
Total plasma, unbound plasma and CSF concentration–time profiles after oral administration of 200 mg tedizolid once daily (Patient 1) or 200 mg tedizolid twice daily (Patient 2). Filled circles correspond to observed concentrations and solid lines represent individual concentrations predicted by the PK model.

Only the unbound fraction (fu) of tedizolid in plasma, whose value (15.7%) was consistent with previous reports,^16^ was assumed to distribute into CSF while protein binding in CSF was considered negligible due to the low concentrations of blood-derived proteins, particularly albumin.^18^ The model-derived unbound peak concentration (*C*_max_) in CSF was ∼2.5 times lower, smoother and delayed compared with unbound plasma; elimination from CSF was also slower (Figure 1). Bidirectional blood-CSF transfer was characterized by clearances into (CL_CSF,in_) and out (CL_CSF,out_) of the CSF, with a CL_CSF,in_/CL_CSF,out_ ratio of 63%, matching the modelled CSF-to-plasma free AUC ratio derived from the model (Tables 1 and S1).

**Table 1.** Model-derived pharmacokinetic pharmacokinetic parameters after oral administration of 200 mg once daily (Patient 1) or twice daily (Patient 2) of tedizolid

	Patient 1				Patient 2			
	AUC_0–24_ (mg/L. h)	*C*_max_ (mg/L)	*T*_max_ (h)	*T*_1/2_ (h)	AUC_0–24_ (mg/L. h)	*C*_max_ (mg/L)	*T*_max_ (h)	*T*_1/2_ (h)
Total plasma	19.0	1.87	2.50	6.33	29.6	1.64	2.50	9.60
Unbound plasma	2.99	0.293	2.50	6.33	8.92	0.493	2.50	9.60
CSF	1.89	0.116	7.30	8.22	5.63	0.254	5.80	11.4

Given the Nocardia MIC of 0.75 mg/L, the *f*AUC_0–24_/MIC ratios for unbound tedizolid in plasma and CSF were 3.99 and 2.25, respectively, indicating potentially subtherapeutic CSF levels. Based on this, tedizolid was discontinued and linezolid resumed at 600 mg three times daily. Despite these changes, the patient’s condition did not improve, and, in accordance with his wishes and family consent, active treatment was withdrawn. The patient died shortly thereafter.

### Case 2

A 72-year-old man with diabetes and a previous stroke presented with fever, confusion and mild dysphasia. Brain MRI revealed multiple abscesses. Biopsy confirmed *N. farcinica* infection (tedizolid MIC: 0.75 mg/L). Initial treatment included imipenem, amikacin and cotrimoxazole. Due to uncertain cotrimoxazole susceptibility, linezolid and minocycline were started, then switched to oral tedizolid because of gastrointestinal side effects and thrombocytopenia.

Given concerns about subtherapeutic CSF concentrations observed in Patient 1, the tedizolid dose was doubled to 200 mg every 12 hours after 9 weeks. Amikacin was discontinued due to hearing loss, and definitive therapy included intravenous imipenem and oral tedizolid. By month 3, imipenem was replaced by cotrimoxazole and later levofloxacin due to intolerance, with MRI showing marked improvement.

At month 4, plasma samples were collected before and at 3, 6 and 12 hours after tedizolid administration; a single CSF sample was obtained at 10 hours via lumbar puncture (Figure 1). Sample processing and assay were identical to Patient 1. The same PK model structure was used, but CSF distribution parameters were fixed to values from Patient 1 due to limited data. However, as only a single CSF measurement was obtained for this patient, the parameters characterizing the cerebral distribution of tedizolid were set to the values estimated for Patient 1, assuming the same penetration rate between the two patients (i.e. identical fAUC_CSF_/*f*AUC_plasma_ ratio). At the time of sampling no other medication was supposed to impact the concentration of tedizolid.

The unbound plasma fraction for Patient 2 was nearly double that of Patient 1 (30.1% versus 15.7%), contributing to a more extensive CNS distribution. The *f*AUC_0–24_/MIC ratio in CSF was 7.5, well above the target of 3. Owing to cytopenia, the tedizolid dose was reduced to 200 mg once daily and stopped at month 7. Two years later, the patient remained well with no recurrence and normal MRI.

## Discussion

We present two cases of CNS nocardiosis caused by *N. farcinica* strains with MICs of 0.75 mg/L, in which tedizolid was included in the antibiotic regimen and pharmacological parameters were obtained.

Tedizolid CSF distribution was moderate (63%) compared with drugs that cross by passive diffusion, such as linezolid, but higher than ceftaroline or meropenem, which have penetration ratios below 20%. The higher CSF efflux than influx clearance suggests that tedizolid may be a substrate for active transporters, limiting its CSF penetration, consistent with preclinical data implicating P-gp and BCRP efflux pumps.^19^

Obtaining rich CSF data is challenging, as illustrated by the single CSF measurement in Patient 2. As a result, we assumed that the cerebral distribution was identical for both patients even though many factors, particularly neuroinflammation, are known to influence this parameter, leading to interindividual variability in drug distribution. Despite this limitation, CSF tedizolid concentrations were predicted adequately in both patients using a model based on individual unbound plasma concentrations and a common CSF penetration rate (Figure 1). This suggests that measuring unbound plasma concentrations is essential for estimating brain exposure, although additional data are needed to confirm this.

Most PK studies estimate unbound plasma concentrations from total concentrations, which may bias the free PK/PD index. Our data showed significant interpatient variability in tedizolid fu, which doubled between patients. Assuming the same fu value for both patients (e.g. 15.7%) would have led to an underestimation of unbound tedizolid concentration in Patient 2, reducing the calculated *f*AUC_0–24_/MIC ratio from 7.5 to 3.9, and could have resulted inappropriate dosing decisions, risking toxicity or therapeutic failure. Therefore, direct measurement of unbound drug concentrations is crucial for optimizing antimicrobial therapy, as opposed to relying solely on estimated fu values, as they do not always accurately reflect true free drug levels in individual patients.

In Patient 1, 200 mg tedizolid once daily did not achieve the CSF PK/PD target, unlike Patient 2 who received a doubled dose. Tedizolid has demonstrated good safety even at higher doses (above 200 mg/day), making it a viable alternative to linezolid for minimizing haematological toxicity. An initial dose of 200 mg every 12 hours may be considered to maximize CSF exposure while awaiting unbound plasma measurements, with subsequent dose adjustment as appropriate.

Although successful CNS nocardiosis treatment with tedizolid has been reported, these cases lacked CSF measurements and involved multiple antibiotics, precluding firm conclusions about tedizolid’s CSF diffusion or efficacy.^6,10^ Its use in CNS infections should be cautious, with systematic MIC determination. Indeed, a limitation of this study is that tedizolid concentrations in CSF may not fully reflect concentrations in brain parenchyma. Nevertheless, previous preclinical data suggest that CSF concentrations may approximate brain extracellular fluid concentrations within a reasonable margin and may therefore serve as a clinically useful, albeit imperfect, surrogate when direct brain measurements are not feasible.^20^

The EUCAST recommends reporting tedizolid as susceptible for all linezolid-susceptible *Streptococcus* and *Staphylococcus* isolates.^4^ However, in the absence of established clinical breakpoints for tedizolid, both for *Nocardia* species and for CNS infections—including meningitis—tedizolid may be considered as a therapeutic option but should be used cautiously until such breakpoints are formally defined.

In conclusion, diffusion differences between linezolid and tedizolid suggest these drugs are not interchangeable for CNS infections. Further studies on tedizolid CSF penetration and CNS infection efficacy are warranted but may be difficult to conduct.

## Supplementary Material

dlag004_Supplementary_Data

## Supplementary data

Figure S1 and Table S1 are available as Supplementary data at *JAC-AMR* Online.
