# Correction: Optimizing Tedizolid Dosing in Cerebral Nocardiosis: Clinical Impact of Direct Unbound Concentration Measurement and Population PK Modelling in Two Cases

**DOI:** 10.1093/jacamr/dlag230

**Published:** 2026-10-27

This is a correction to: Marc-Olivier Vareil, Margaux Bouet, David Leyssene, Anne-Christine Jaouen, Heidi Wille, Christophe Adier, Laure Alleman, Alexia Chauzy, Optimizing Tedizolid Dosing in Cerebral Nocardiosis: Clinical Impact of Direct Unbound Concentration Measurement and Population PK Modelling in Two Cases, *JAC-Antimicrobial Resistance*, Volume 8, Issue 1, February 2026, dlag004, https://doi.org/10.1093/jacamr/dlag004

In the originally published version of the paper the given and last names of the second to the last co-authors were erroneously transposed. These are now in the correct order. The publisher apologizes for this error.

