# Supplementary material for: Optimizing Tedizolid Dosing in Cerebral Nocardiosis: Clinical Impact of Direct Unbound Concentration Measurement and Population PK Modelling in Two Cases
Source: JAC Antimicrob Resist. 2026 Jan 29;8(1):dlag004. doi: 10.1093/jacamr/dlag004 (PMC12852995; doi:10.1093/jacamr/dlag004)
Supplement: dlag004_Supplementary_Data [file dlag004_supplementary_data.docx]

**Supplementary data**


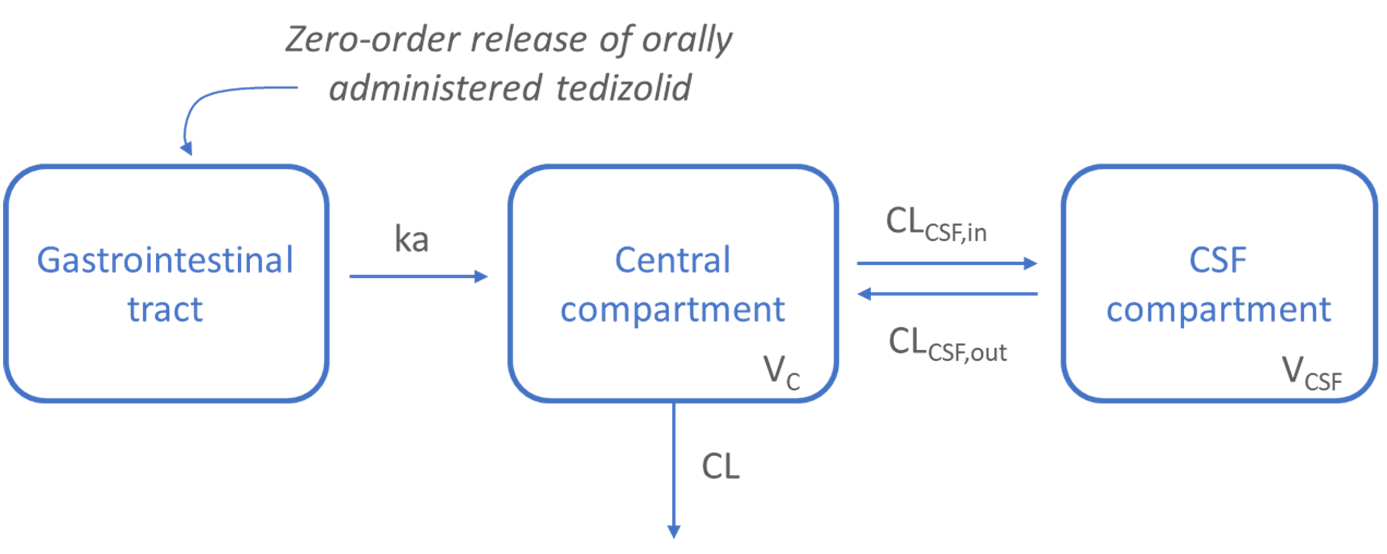


Figure S1. Schematic representation of the final PK model for oral administration of tedizolid. CL corresponds to the clearance of central compartment; VC and V_CSF_ correspond to volumes of central and CSF compartments, respectively; CL_CSF,in_ and CL_CSF,out_ are the CSF input and output clearances respectively; ka is the first-order absorption rate constant.

| Table S1. Parameter estimates of the final pharmacokinetic models for tedizolid | | | | | | |  |
| --- | --- | --- | --- | --- | --- | --- | --- |
|  |  | **Patient 1** | |  | **Patient 2** | |  |
| **Parameter** | **Definition** | **Estimate** | **95%CI** |  | **Estimate** | **95%CI** |  |
| DUR (h) | Duration of zero-order absorption | 1.62 fixed |  |  | 1.62 fixed |  |  |
| Ka (h^-1^) | First-order absorption rate constant | 1.99 fixed |  |  | 1.99 fixed |  |  |
| CL (L/h) | Clearance of central compartment | 10.5 | [8.87 – 12.3] |  | 13.5 | [12.9 – 14.1] |  |
| V_C_ (L) | Volume of central compartment | 95.8 | [75.1 – 125] |  | 187 | [164 – 222] |  |
| fu | Unbound fraction in plasma | 0.157 | [0.132 – 0.192] |  | 0.301 | [0.291 – 0.312] |  |
| V_CSF_ (L) | Volume of CSF compartment | 0.15 fixed |  |  | 0.15 fixed |  |  |
| CL_CSF,in_ (L/h) | CSF input clearance | 0.0193 | [0.0111 – 0.0323] |  | 0.0193 fixed |  |  |
| CL_CSF,out_ (L/h) | CSF output clearance | 0.0306 | [0.0192 – 0.0450] |  | 0.0306 fixed |  |  |
| σ_plasma, total_ (%) | Proportional residual variability for total plasma | 14.2 | [10.8 – 19.0] |  | 4.12 | [2.92 – 5.35] |  |
| σ_plasma, free_ (%) | Proportional residual variability for free plasma | 24.9 | [18.1 – 30.9] |  | 6.44 | [4.50 – 8.89] |  |
| σ_CSF_ (%) | Proportional residual variability for CSF | 19.3 | [14.2 – 26.0] |  | **-** |  | |
| The 95% CI was obtained by Sampling Importance Resampling (SIR).  Parameters fixed due to identifiability issues to:   1. The parameter values from a previous model developed by Flanagan et al. 2. The physiological volume of CSF 3. The values estimated from data from patient 1 | | | | | | |  |
